# Integrating behaviour, physiology, and immune response to social stress in fish

**DOI:** 10.3389/fendo.2026.1743867

**Published:** 2026-04-15

**Authors:** Svante Winberg, Pavla Hubená

**Affiliations:** 1 Behavioral Neuroendocrinology, Department of Medical Cell Biology, Uppsala University, Uppsala, Sweden; 2 Department of Animal Biosciences, Swedish University of Agricultural Sciences, Uppsala, Sweden

**Keywords:** aggression, coping, immune response, proactive, reactive, stress, transcriptomics

## Abstract

Dominance hierarchies in fish are established through conflicts and lead to significant differences in stress physiology, behaviour, and immune function between dominant and subordinate individuals. This review explores the relationship between social stress within these hierarchies, individual variations in stress responses and cognitive bias towards stressful situations, and the impact of conflicts on future performance and interactions between stress and the immune system. Fish express divergent stress coping styles (proactive and reactive) that differ in the reactivity of the stress axes ending with the release of the major stress hormones (adrenaline and cortisol). Social stressors, like conflicts, are perceived differently by proactive and reactive fish, leading to varying levels of stress hormone release. The stress hormones interact with the immune system, changing individuals’ ability to fight off pathogens. Pro-inflammatory cytokines highly activated in reactive individuals under stress can provide feedback to the monoaminergic system in the brain, resulting in depression-like, anxiety-like, or “sickness” behaviour. The review also discusses strategies for reducing social stress in fish and enhancing their overall health in aquaculture, while emphasising the importance of considering these factors in research settings to prevent data bias.

## The stress response

Stress is a complicated concept that was originally described as a non-specific response of the animal to threats to homeostasis and survival (1, 2). The stress response is primarily driven by the activation of the sympathetic nervous (SNS) system and the release of glucocorticoids impacting various physiological processes such as respiration, circulation, and metabolic processes (3–5). During threatening situations, the physiology of the animal is shifted to a catabolic state, making energy substrates available for fight and flight response (4). While traditionally seen as a non-specific reaction, recent research indicates that the stress response is influenced by the nature of the challenge, past experiences, environmental context, genetic factors (stress coping style and personality), and sex (6, 7). In addition to the physiological effects of stress, challenges can also impact behaviour, other bodily systems, and potentially even future generations through epigenetic mechanisms (8). While considerable research has been done on stress in mammals, there is a lack of knowledge regarding stress responses in teleost fish and other non-mammalian vertebrates (9, 10). However, emerging evidence suggests that stress responses are similar across different species, highlighting the evolutionary conservation of these mechanisms (11–13). This review will explore the physiological and behavioural responses to social interactions, variations in these responses within species, the long-term impact of social experiences on health, including immune function, and the intricate relationship between stress responses and immune functions, focussing particularly on teleost fishes.

## Social hierarchy in fish

Teleost fish represent a diverse group of vertebrates, but it is important to note that the discussion in this review is focussed on specific species rather than all species. In certain fish species, social systems can be based on monogamous pair-bonds, harems, shoaling, or schooling with group spawning (14). However, the focus of this review is on group-living teleosts commonly used in laboratories and aquaculture globally, such as zebrafish and salmonids, which establish dominance-based social hierarchies (7). This distinction is crucial as the mechanisms discussed may not apply universally to all species. Living in a dominance-based social hierarchy offers benefits like shoaling, schooling, and mating opportunities, but maintaining one’s position within the hierarchy involves repeated conflicts that lead to social stress, a unique stressor not typically found in non-social species. Research suggests that the structure of dominance hierarchies can vary between species or even among populations of the same species. For instance, the dominance hierarchy of rainbow trout (*Oncorhynchus mykiss*) may be either despotic (dominated by a single individual) or oligarchical (with a small group of dominating individuals) based on individual food intake (15). In such hierarchies, other individuals in the group typically follow a linear order, with a few individuals occupying lower ranks. Fish are capable of inferring social rank by observing interactions between other individuals, a phenomenon known as transitive inference (16). This ability allows fish to understand the hierarchical order even in the absence of direct conflicts. Individual positions within the hierarchy are established through agonistic behaviours, including overtly aggressive acts like biting and chasing, which can lead to increased risks of injury and stress in fish (17).

There are significant behavioural and physiological variances between fish occupying different social ranks. The formation of a hierarchy typically induces high levels of stress for all individuals involved, including the most competitive fish that emerge victorious in aggressive interactions and establish dominance (7, 18, 19). However, once the hierarchy is firmly established, dominant fish often exhibit no signs of stress as long as the hierarchy remains stable (20, 21). These dominant individuals tend to initiate further challenges to reaffirm their position and trigger physiological responses linked to the hierarchy, such as the release of 11-ketotestosterone and androgens. This is known as the “winner effect” (22–24). Conversely, social subordination results in a prolonged activation of stress responses, behavioural inhibition, and additional physiological consequences of stress (20). Subordinate individuals are known to reduce or completely forgo food consumption, avoid conflicts, and display weaker secondary sexual characteristics—known as the “loser effect” (22–24). Abbott et al. (25) have shown that in juvenile rainbow trout (*Oncorhynchus mykiss*), the impact of previous victories far outweighs body size effects, a trend also seen in other teleosts such as zebrafish (22, 26). The loser effect is likely associated with behavioural inhibition influenced by the prolonged activation of the brain’s serotonergic system and other neuroendocrine stress effects (18, 19). In a stable hierarchy, the costs of conflicts diminish for dominant fish, whereas subordinates tend to avoid retaliation. Disrupting an established social hierarchy by removing the dominant individual results in rapid behavioural changes in second-ranked fish, driven by the potential for elevation in the hierarchy, which can be even more pronounced in fish undergoing sex changes (27). Individuals strive to enhance their position within the social hierarchy to gain greater access to resources.

Dominance hierarchies within a single species can be influenced by the environment. Using terms from sociology, fish populations can exhibit dominance hierarchy of variable intensity ranging from egalitarian to stratified. A strong dominance hierarchy, or a stratified population, can occur when fish density is low (less than or equal to one fish per litre in zebrafish) and resources are scarce or defendable (28, 29). This results in significant physiological and behavioural differences between dominant and subordinate fish due to social stress and reduced access to resources for subordinates. In contrast, egalitarian populations have fewer aggressive interactions between fish and fewer physiological differences between individuals. This occurs when resources are equally available and the population is large enough (at least three fish per litre in zebrafish) (28). The effect of the fish density may be explained by the fact that small-sized groups are almost always linear, whereas the number of intransitive triads is more likely to increase with the increasing number of individuals (30). This theory was supported by the work of Bessa et al. (31) that showed that individuals from low-density groups had larger differences between individuals in basal cortisol release compared with groups kept at high stocking density (31). Overall, environmental factors can influence social hierarchies and minimise differences between individuals within a population despite competition for resources and social dominance.

## Neuroendocrine responses to social stress

The physiological response to acute stress experienced during the establishment of social hierarchies or in response to hierarchical instability is primarily driven by the activation of the sympatho-chromaffin system and the hypothalamic–pituitary–interrenal axis (HPI axis, equivalent of the mammalian hypothalamic–pituitary–adrenal axis), in addition to behavioural arousal. Social stress has been found to alter brain monoaminergic activity in teleosts and other vertebrates. The activation of brain monoaminergic systems, such as the norepinephric (NE), dopaminergic (DA), and serotonergic (5-HT) systems, occurs rapidly during the establishment of dominance hierarchies (19, 21). While winners see a return to control levels of brain 5-HT activity once the hierarchy is established, losers exposed to chronic social stress often exhibit elevated levels of 5-HT activity. Some studies have also found that NE activity remains elevated in subordinate fish after prolonged social interaction, whereas an increase in forebrain DA activity seems to be more related to the acute stress and behavioural arousal (21, 32, 33). The chronic elevation of brain 5-HT activity in subordinates, particularly in forebrain and hindbrain areas, has been identified as a mechanism that mediates the observed behavioural inhibition in these individuals (18, 19). However, acute activation of the brain 5-HT system does not lead to behavioural inhibition, highlighting the importance of the duration of the stressful challenge.

The reasons behind why sustained increase in brain 5-HT activity leads to reduced behavioural responsiveness are not yet fully understood. One theory suggests that a sudden activation of the DA system may counteract the inhibitory effects of 5-HT, as DA has behavioural effects that oppose those of 5-HT. Another possibility is that prolonged elevation of synaptic 5-HT release triggers neuroplastic responses that influence behavioural responses over a longer period of time. Therefore, the social status of an animal significantly influences its physiology, behaviour, and life history. This is particularly evident in teleosts, a group of vertebrates known for their adaptability (34). But which factors determine animal’s success in conflict and ability to attain a high rank in the dominance hierarchy?

## Stress coping styles and effects on the outcome of fights for social dominance

Differences among individuals within a single population were historically considered as a “noise” until the introduction of standardised ethological assessments in the 20th century. Réale et al. in 2007 established a framework for assessing animal personality by identifying five main behavioural traits: activity, aggression, boldness, exploration, and sociability (35). This study identified the five main behavioural traits: activity, aggression, boldness, exploration, and sociability. Each trait represents a spectrum from low to high (e.g., from inactive to highly active in the case of activity). Each individual displays a relatively consistent level of these traits in standardised tests (35). Correlations between different behavioural traits were observed, leading to the identification of “behavioural syndromes,” such as the correlation between activity and boldness levels (36). Studies further revealed correlations between a range of behavioural traits and specific stress responses known as stress coping styles (**Table 1**), which remain stable across situations and lifespan (10, 76, 77). Proactive animals exhibit traits such as boldness, exploratory behaviour, and aggression, with stress responses characterised by strong sympathetic activation and modest activation of the HPI/HPA axis. In contrast, reactive animals display contrasting behavioural and neuroendocrine profiles. Stress coping styles have also been linked to the pace of life syndrome (POLS) in Atlantic salmon (*Salmo salar*), which proposes that consistent behavioural traits across time and contexts are interconnected with life-history traits (45). Fast pace of life is associated with rapid development, bold and aggressive characteristics, and premature mortality compared with a slow pace of life (78). Research on Atlantic salmon indicated that proactive individuals are more prevalent among those with a faster pace of life (45). The behavioural and physiological differences between proactive and reactive fish also impact their susceptibility to diseases. For instance, rainbow trout with higher ventilation volumes are more likely to be infected by trematode cercariae, which tend to grow larger in faster-growing fish, making proactive individuals more susceptible to this condition (79, 80). On the other hand, reactive fish are particularly vulnerable to bacterial and viral infections (81, 82). These variations in stress coping styles across multiple levels have implications for life history and resilience to environmental challenges.

**Table 1:** Outline of genetic, endocrine, and behavioural features of stress coping styles in fish with expected outcomes of the differences.

Genetic background	Endocrine background	Behavioural background	Expected outcome
Proactive			
*Genes*	*CNS response*		*Metabolism*
(−/+) *crh1r* (37–39)	(+) sympathetic reactivity (40)	(+) aggression (37, 41–44)	Fast pace of life (45)
(+) *fthl29* (37)	(−) HPI axis activity (46, 47)	(+) boldness (37, 41, 42, 46, 48–54)	(+) mobilisation speed of resources and oxygen
(+) *tfr1a* (37)	(−) HPI axis reactivity (38, 43, 44, 48–51, 55–58)	(+) exploration (37, 42, 50, 55)	(+) metabolic rate (59)
(+) *slc11a2* (37)	(−) 5-HT, NE, DA turnover in BS and OT (60, 61)	(+) activity (37, 42, 48, 50, 51, 53, 58, 62)	(−/+) oxygen consumption (63)
(+) *bdnf* (38)	(+) 5-HT and NE metabolites in HT (60)	(+) feeding resumption (47, 55, 62)	(+) gill branching (64)
(+) *5-HT1A* (38)	(+) 5-HT turnover in brain (38, 60)	(−/+) memory retention (41, 49, 58, 65)	(+) stress recovery speed (53, 66, 67)
(+) *gapdh-2* (68)	(+) 5-HIAA post stress (60)	(+) escape attempts duration (47)	(+) body size (69)
(+) *gr1* (61)	(−) NE (39, 46)		(+) body pigmentation (70)
(+) *mr* (61)	*Peripheral response*		*Immune response*
(+) *mr/gr1* (61)	(+) intestinal metabolism (69)		(+) immunity (69)
*Processes*	(+) splenocyte response (69)		Altered iron metabolism in brain (37)
(+) protein metabolic process (71)	(+) 11-ketotestosterone (41, 52)		*Behavioural response*
(+) regulation of cell cycle (71)	(−) lactate (46)		Routine (41, 72, 73)
(+) ribosomal proteins (71)	(+) IgM (53)		Optimism (55)
(−) neuroplasticity (37)	(+) NK cell activity (69)		(+) chance of winning (43)
	(−) cytokine production (69)		*Reproduction*
			Coercive mating strategy (54)
			(+) yolk size at emergence (74)
			(+) larvae emergence speed (67, 74)
			(+) number of offspring (51)
Reactive			
*Genes*	*CNS response*		*Metabolism*
(−/+) *crh1r* (37–39)	(−) sympathetic activity (40)	(−) aggression (37, 41–44)	Slow pace of life (45)
(+) *viml* (37)	(+) HPI axis activity (46, 47)	(−) boldness (37, 41, 42, 46, 48–54)	(−) mobilisation speed of resources and oxygen
(+) *tlr7* (37)	(+) HPI axis reactivity (38, 43, 44, 48–51, 55–58)	(−) exploration (37, 42, 50, 53, 55)	(−) metabolic rate (59)
(+) *pcna* (38, 57)	(+) 5-HT, NE, DA turnover in BS and OT (60, 61)	(−) activity (37, 42, 48, 50, 51, 53, 58, 62)	(−/+) oxygen consumption (63)
(−/+) *mr* (39, 61)	(−) 5-HT and NE metabolites in HT (60)	(−) feeding resumption (47, 55, 62)	(−) gill branching (64)
(+) *gr2* (39, 75)	(−) 5-HT turnover in brain (39, 60)	(−/+) memory retention (41, 49, 58, 65)	(−) stress recovery speed (53, 66, 67)
(+) *egr1* (39)	(+) NE (39, 46)	(−) escape attempts duration (47)	(−) body size (69)
(+) *neurod2* (39, 57)	*Peripheral response*		(−) body pigmentation (70)
(+) *dcx* (57)	(+) intestinal muscle activity (69)		*Immune response*
(−/+) *gapdh* (68, 75)	(+) intestinal regeneration (69)		(−) immunity (69)
(−) *gr1 (*61)	(+) intestinal immune response (69)		(+) intestinal inflammation risk (69)
(−) *mr/gr1 (*61)	(+) gut-brain axis (69)		(+) neuroinflammation risk (37)
*Processes*	(−) splenocyte response (69)		(+) neurotoxicity risk (37)
(+) synaptic transmission (71)	(+) lactate (46)		
(+) response to gonadotropin (71)	(−) IgM (53)		*Behavioural response*
(+) response to purine compounds (71)	(−) NK cell activity (69)		Adaptive (41, 72, 73)
(+) cell adhesion (71)	(+) cytokine production (69)		Pessimism (55)
(+) transmembrane transporter activity (71)			(−) chance of winning (43)
(+) neuroplasticity (37)			*Reproduction*
			Courting mating strategy (54)
			(−) yolk size at emergence (74)
			(−) larvae emergence speed (67, 74)
			(−) number of offspring (51)

5-HIAA, 5-hydroxyindoleacetic acid; 5-HT, 5-hydroxytryptamin; BS, brainstem; DA, dopamine; HPI axis, hypothalamo–pituitary–interrenal axis; HT, hypothalamus; IgM, immunoglobulin M; NE, norepinephrine; NK cell, natural killer cell; OT, optic tectum. The symbols in brackets signify the direction of change found in literature: + = increase, − = decrease, +/− both increase and decrease were found.

Positions within the dominance hierarchy are determined by pairwise agonistic interactions, with body size and physical strength playing crucial roles (7, 17). However, factors related to behavioural phenotype and aggressiveness may outweigh body size differences in determining hierarchy positions, if differences in body size between contestants is not large (83). Do individuals with different stress coping styles vary in their response to social challenges, such as fights for social dominance? Proactive animals, known for their aggressiveness, often have an advantage in establishing dominant-subordinate relationships. This trend has been observed in various species as was found in three-spined sticklebacks (*Gasterosteus aculeatus*), rainbowfish (*Melanotaenia duboulayi*), anemonefish (*Amphiprion clarkii*), zebrafish, and rainbow trout (43, 83–86). In contrast, previous experience seems to be a better predictor of dominance in mangrove killifish (*Kryptolebias marmoratus*), unique for its preferential self-fertilisation (87). Proactive individuals also respond to challenges with a stronger sympathetic activation than reactive individuals, which is another factor that may give them an advantage in agonistic interactions as stronger sympathetic activation will result in faster and stronger mobilisation of metabolic resources and oxygen supply. In addition, differences in response to winning or losing fights may occur between stress coping styles, which may potentially affect competitiveness in dominance struggles, possibly linked to the appraisal of these challenges.

Appraisal, cognitive bias, and stress coping styles are potentially interlinked factors. Appraisal is the process of evaluating situation base on multiple feats that differ across studies, such as controllability, novelty, and familiarity (88). The resulting assessments of these appraisals manifest as emotions detected through physiological changes and behaviour (89). For example, encountering a predator is assessed as a sudden uncontrollable threat, which increases fight/flight/freeze response, heart rate, and vigilance. This response is primarily driven by the sympathetic nervous system and is associated with fear emotion (89). The amygdala plays a crucial role in processing fear emotions, with its activity regulated by the prefrontal cortex (90). High sympathetic tone reduces the PFC regulatory ability over amygdala, which heightens its metabolic activity (90). Moreover, past experiences can shape future appraisals, as seen in Pavlovian fear conditioning where a neutral cue associated with a negative event can elicit similar behavioural and physiological reactions later on (91). Appraisal theory states that it is not just the intrinsic characteristics of the stressor that determines the magnitude of the stress response. Instead, the stressor is judged based also on the state of the individual (e.g., needs, resources, and experience). Consequently, responses to stressors can vary among individuals (cognitive judgement bias) and can even differ based on the context in which the stressor is encountered (see (13) and (12) for a review). In other words, individuals with varying past experiences may interpret the same situation differently.

**Figure 1:**
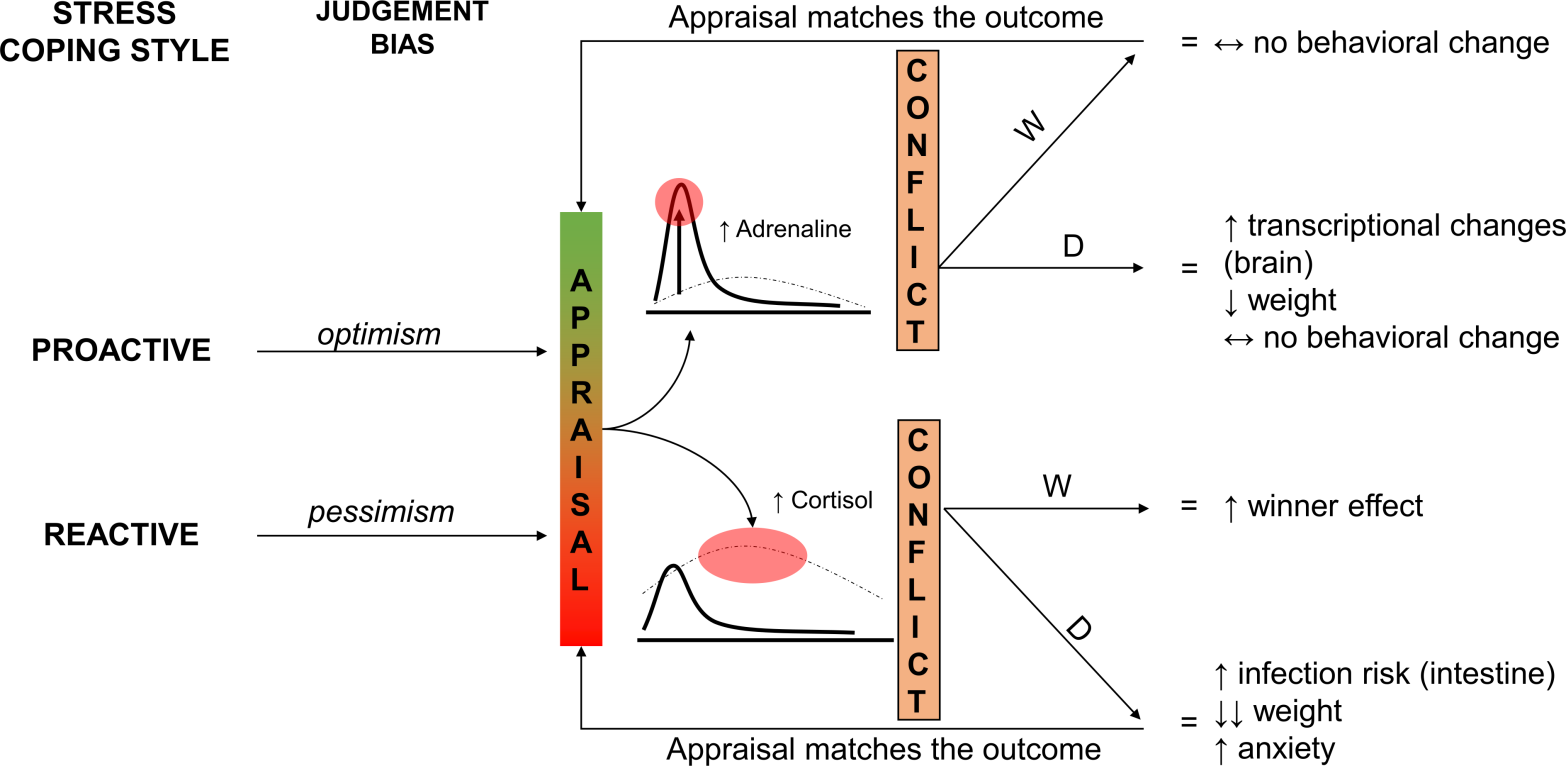
Schematic image of the consequence of the judgement bias on the outcome of a contest in proactive and reactive fish (W, win; D, defeat). See Espigares et al. (94), Hubená et al. (37), and Benrejdal et al. (69) for more details.

Cognitive judgement biases in decision-making have been observed in various vertebrates, including teleosts (92). Research has shown that some individuals tend to interpret ambiguous stimuli in a positive manner (optimism), whereas others may view the same stimuli as negative (pessimism). Studies have primarily focussed on how affective states can influence judgement bias (92). It has been found that stress and environmental challenges can lead to a more negative interpretation of ambiguous stimuli (pessimism) (93). Recent research has also suggested a potential link between cognitive judgement bias (optimism or pessimism) and personality traits (93). It has become increasingly evident that fishes, like other vertebrates, exhibit subjective appraisal, indicating that fishes may possess cognitive judgement bias, where some individuals perceive a stimulus positively whereas others interpret the same stimulus negatively (94). Espigares et al. (94) conducted a study on adult male zebrafish and identified a bimodal distribution of positive (optimistic) and negative (pessimistic) phenotypes in terms of judgement bias. They also found that cognitive judgement bias is a personality trait linked to boldness, with optimistic males displaying greater boldness compared with pessimistic males. Thus, it seems that positive cognitive judgement bias, being optimistic, is part of the proactive stress coping style, whereas being pessimistic, showing negative cognitive bias, is part of the reactive behavioural profile. This suggestion is supported by the fact that in the study by Espigares et al. (94), optimists and pessimists were found to differ in stress responses and HPI axis control. Positive judgement bias in proactive fish could influence how they react to winning or losing agonistic interactions (**Figure 1**).

In a recent study, males from two zebrafish lines selectively bred for bold and shy behaviour in the novel tank diving test were examined (37). The study focussed on the effects of social interaction on behaviour and brain transcriptomics. Aggressive behaviour was measured before and after 6 days of dyadic interaction using a mirror test, and the zebrafish multivariate concentric square field (zMCSF) test (95) was used to evaluate behavioural profiles following 3 days of interaction. The results revealed distinct differences in behaviour between the bold and shy fish lines, with bold fish displaying proactive coping whereas shy fish exhibited reactive behaviours. Additionally, the response to social interaction varied between the two lines, with proactive fish being more aggressive initially but showing no change in behaviour after social subordination. In contrast, reactive fish displayed typical loser effect following dyadic interaction. Among fish winning the dyadic interactions, only the reactive winners showed a clear winner effect: increase in aggressive behaviour. Thus, reactive fish were more adaptable in their behaviour based on social experiences, whereas proactive fish displayed more routine-based feedforward control (37). Proactive stress coping has repeatedly been connected with routine-based feed forward behavioural control with limited response to changing environmental stimuli (41). The behavioural profiles assessed by the zMCSF test validated that bold fish were bolder and more exploratory than shy fish, with effects of social interaction observed only in the reactive phenotype (37).

Interestingly, the brain gene expression effects showed an opposite pattern, with proactive fish exhibiting a larger number of differentially expressed genes compared with reactive fish (37). This suggests that reactive fish may be able to mitigate the effects of social stress by adjusting their behaviour, highlighting the role of behavioural flexibility. Previous research has demonstrated that social interaction can impact boldness in juvenile rainbow trout (7, 96).

In summary, the outcome of fights for social dominance in fish is heavily influenced by the behavioural profiles of the individuals involved. Fish displaying proactive stress coping styles tend to have an advantage over those with reactive coping styles. Winning and losing fights have different effects on proactive and reactive fish, with differences possibly related to how these experiences are perceived (cognitive judgement bias). Reactive fish demonstrate greater behavioural plasticity in response to social challenges but may also experience other effects of social stress that could compromise their performance.

## Secondary effects of social stress

Social hierarchy and individual personality traits have been shown to influence the health status of fish. The skin and intestine are two major barriers protecting fish from pathogens, making them essential for overall health and performance. Using males of the zebrafish lines selected for boldness (37)(discussed above), Benrejdal et al. (69) quantified gene expression in skin and intestine of fish from the proactive and reactive male zebrafish with experience of social dominance or subordination. The findings revealed significant differences in intestinal gene expression between proactive and reactive fish. While social status had a modest impact on intestinal gene expression, subordinate reactive fish showed an enrichment of genes related to peptidase activity, higher expression levels of cancer-associated and immunity-associated genes, and lower expression of genes involved in antiviral immunity compared with proactive fish. On the other hand, gene expression in the skin was more influenced by social status, with differential expression of genes related to immunity, stress, and metabolism. This divergence in skin gene expression between dominant and subordinate fish may be attributed to damage inflicted on the skin of subordinates by aggressive attacks from dominant fish. The enrichment of peptidase activity in the intestines of subordinate reactive fish suggests an increased risk of damage and infections in the gastrointestinal tract. Similar results were observed in juvenile rainbow trout, where stress was found to compromise gut integrity in fish exhibiting a reactive coping style characterised by high plasma cortisol levels (97). In the study conducted by Espigares et al., it was found that zebrafish with a pessimistic disposition, likely indicative of a reactive coping style, exhibited a higher rate of tumourigenesis compared with their proactive optimistic counterparts (94). Similarly, in the study by Benrejdal et al. (69), the cancer-related genes were differentially expressed with subordinate fish of the shy line showing the highest expression. Additionally, in common carp (*Cyprinus carpio*), fish with proactive and reactive stress coping styles displayed variations in the expression of immune-related genes under both normal and immune-challenged conditions (98). There is growing evidence to suggest a connection between the autonomous and neuroendocrine systems and the immune system, which may explain the link between stress coping styles and immune responses (99). The autonomic nervous system innervates organs of the immune system and immune cells express receptors for various hormones and regulatory peptides (100, 101). Because of the physiological differences between stress coping styles to stress, it can be anticipated that divergent stress-induced effects on the immune system between proactive and reactive fish is exhibited (99).

## Stress and gut–brain–immune interactions

Our knowledge on the relationship between stress coping and immune functions is limited and ambiguous. Most research on the impact of stress on immune function, and how this impact may vary depending on individual stress coping styles, has been conducted on rodents. For example, studies on rats selected for active avoidance behaviour, resulting in phenotypes resembling proactive and reactive stress coping styles, have shown differences in immune function. Low-avoidance (reactive) animals exhibited lower natural killer cell activity and a reduced response of splenocytes to mitogen stimulation compared with high-avoidance (proactive) rats (102). Similar differences in immune function have also been observed in pigs exhibiting different coping styles (103).

**Figure 2:**
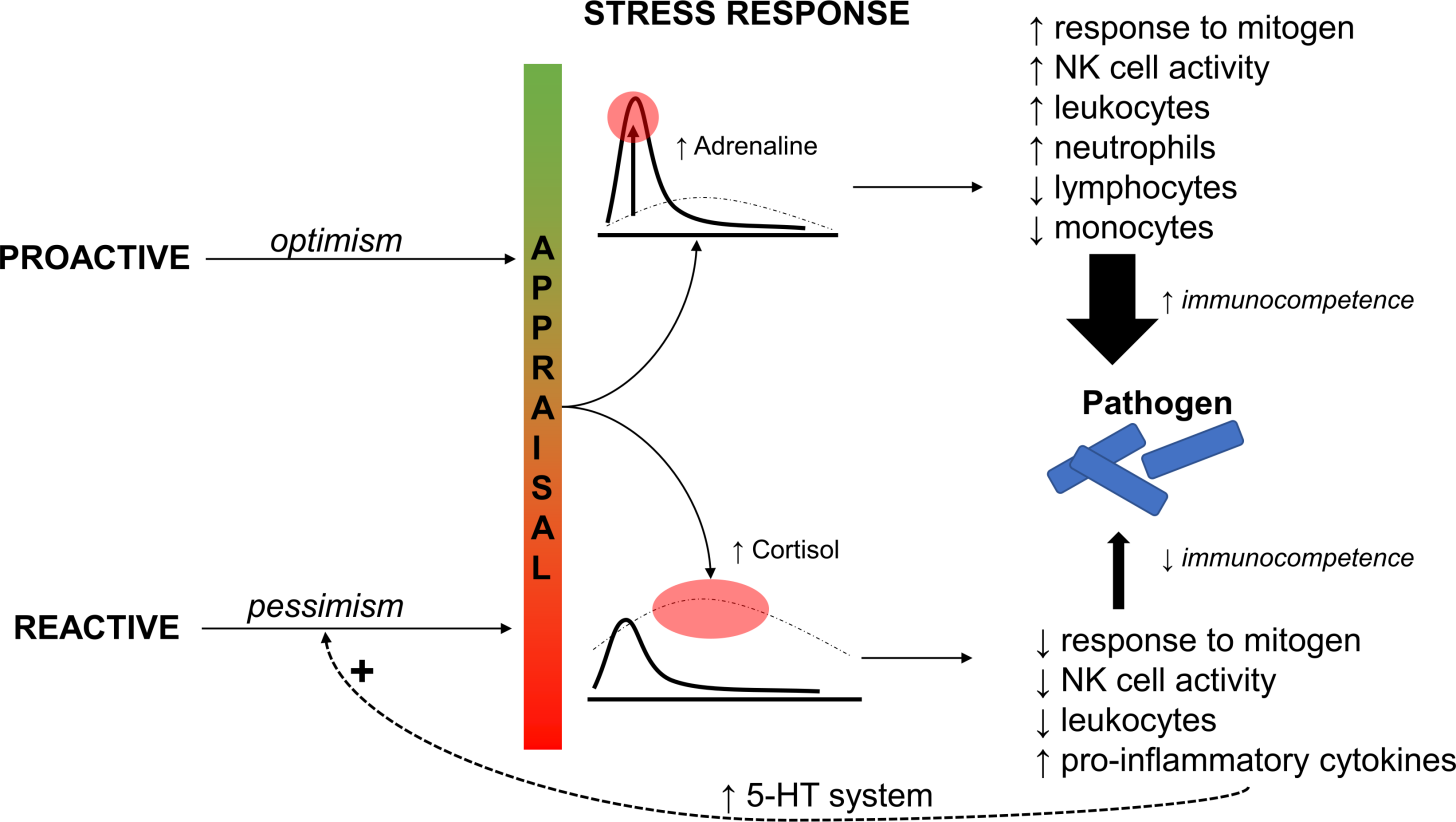
Schematic image showing the link between the cognitive judgement bias, physiological response to stress, effects of the stress hormones on the immune system, response to pathogen (thickness of the arrows indicates the chance of pathogen elimination), and the feedback to the brain through the serotonergic (5-HT) system.

In teleost fishes, as in mammals, the neuroendocrine and immune systems have a close interaction, with stress playing a significant role in affecting immune responses. The impact of stress on immunocompetence can vary, with stressors having both enhancing and suppressive effects depending on their severity and duration. Acute and chronic stressors may act on different mechanisms, sometimes having agonistic effects. In response to acute stress, sympathetic activation leads to an increase in circulating leukocytes, which are then distributed to target tissues (104). This results in a decrease in the percentages of lymphocytes and monocytes, but an increase in the proportions of neutrophils, as observed in marine fish such as mullet (*Mugil cephalus*) (104, 105) (**Figure 2**). In mammals, acute stress can also trigger an increase in T-cell activation and the transfer of surveillance T cells to the skin (106). The primary response of the immune system to stress, mediated by catecholamines, prepares the body to fight off invading pathogens. However, long-term activation of stress responses can lead to immunosuppressive effects, particularly due to the continuous activation of the HPI axis. Chronic stress often results in a decrease in blood leukocyte numbers, which is correlated with the intensity of the stressor (107). The fact that fish displaying divergent stress coping styles differ in their endocrine stress responses, proactive fish respond with a stronger activation of the sympathetic response, and reactive fish respond with a more modest activation of this system but instead a more prominent activation of the HPI response makes it likely that proactive fish show a stronger enhancing effect of stress on the immune system than reactive individuals. In the wild, fish can enhance their immune system effectiveness by engaging in “behavioural fever,” where they move to warmer waters to fight off infections (108). However, this phenomenon is unlikely to be observed in laboratory zebrafish, which are kept at stable temperatures throughout their lifetime.

It is increasingly evident that immune activity and cytokine release in the periphery have an impact on brain chemistry and behaviour (109). Immune challenges, such as lipopolysaccharide (LPS) administration, can lead to elevated levels of pro-inflammatory cytokines in both plasma and brain tissue (109). This elevation is more enduring in the brain and can induce depressive-like behaviours and anxiety, known as “sickness behaviour”. Conversely, the anti-inflammatory cytokine IL-10 can prevent depression-like behaviours triggered by pro-inflammatory cytokines or LPS administration. Additionally, stress and high levels of plasma glucocorticoids can cause an increase in pro-inflammatory cytokines. The interaction between the immune system and the HPA/HPI axis seems to work in both directions as immune activation and pro-inflammatory cytokines can stimulate the HPA/HPI axis (109). The behavioural effects of immune activation and cytokines are likely mediated through the brain’s monoaminergic systems, particularly the serotonergic system. Cytokines may impact monoamine biosynthesis in various ways. For instance, in rodents, the activation of p-38 mitogen-activated protein kinase (MAPK) by interleukin-1 b (IL-1b) and tumour necrosis factor α (TNFα) is linked to increased expression and function of 5-HT reuptake transporters (SERT) (109). This, in turn, leads to reduced extracellular 5-HT levels and increased depression-like behaviours (109). Moreover, inflammation can generate reactive compounds like reactive oxygen species (ROS) and nitrogen species (RNS) that may interact with tetrahydrobiopterin (BH4), a cofactor for enzymes involved in monoamine neurotransmitter biosynthesis, thereby reducing the rate of monoamine synthesis. Another important mechanism is the activation of the enzyme indoleamine-2,3-dioxygenase (IDO), which diverts the metabolism of L-tryptophan towards kynurenine instead of 5-HT. This results in decreased brain 5-HT levels and an increase in quinolinic acid, a neurotoxic metabolite (109).

Our understanding of the impact of immune activation and cytokines on brain functions and behaviour has primarily come from studies on rodents. However, a recent study indicates that these effects, along with the underlying mechanisms, are also present in zebrafish, suggesting evolutionary conservation. Mojzesz et al. (110) demonstrated that infection with tilapia lake virus triggered microglial activation, neuroinflammation, and “sickness behaviour” in zebrafish. The inflammatory responses observed, such as changes in cytokine profiles and increased expression of microglia/macrophage markers, closely resembled those seen in mammals. Furthermore, the influence of stress on brain and immune functions may involve the gut microbiota. There is a bidirectional relationship between gut microorganisms and the brain, encompassing neural, endocrine, metabolic, and immunological pathways, as observed in mammals (111). Similar communication systems between the gut and brain also appear to exist in zebrafish (112). The gut microbiota impacts brain development and neural plasticity by modulating brain-derived neurotrophic factor (BDNF) levels and providing neurotransmitters like GABA, 5-HT, and NE. It is crucial for priming the immune system in both the periphery and the brain. Additionally, microbiota influence tryptophan metabolism, creating an additional means of communication between the gut and brain since tryptophan serves as the precursor for 5-HT synthesis in the brain, with the availability of tryptophan limiting 5-HT production.

## Summary and future perspectives

From being considered a general, non-specific response to any threat to the homeostasis of an individual, it is becoming increasingly clear that stress responses differ between individuals as well as between contexts. Appraisal mechanisms, similar to those described in mammals, have been demonstrated in teleosts and other non-mammalian vertebrates, leading to optimistic and pessimistic individuals responding differently to the same challenge (94). Moreover, cognitive judgement bias appears to be part of the divergence in stress coping styles, with proactive animals being optimistic whereas reactive individuals are more pessimistic, explaining why proactive animals may have an advantage in fights for social dominance due to their confidence in their fighting ability (94). As a result, the stability of the behavioural profile may differ between coping styles, with proactive animals having a more stable profile, whereas reactive animals are generally more plastic in their behaviour. These findings are relevant for aquaculture and the use of fishes as vertebrate model organisms in biomedical research.

The domestication of fish is known to lead to a more proactive phenotype, especially in aquaculture where selective breeding programs are utilised to enhance growth and increase the fish’s ability to withstand challenges. Traits such as higher metabolic rate, increased growth potential, lower post-stress cortisol levels, and a more routine-based behaviour are associated with proactive stress coping, which can have beneficial effects on aquaculture productivity (**Table 1**). However, aggressive behaviour in proactive fish can have negative consequences, as it can result in the development of strong dominance hierarchies leading to heightened social stress. In salmonids, highly aggressive fish tend to have higher body mass as dominant individuals, whereas subordinate fish may have lower body mass due to decreased feed consumption, even when provided in excess (15). Proactive fish also exhibit rigid behaviour and struggle to adjust their behaviour in response to social subordination (37), potentially increasing stress levels and mortality rates. Reducing the strength of social hierarchies among fish is essential to improving feed consumption among subordinate individuals. Furthermore, selecting for proactive fish may increase the population’s susceptibility to certain diseases such as parasitism while reducing the occurrence of viral and bacterial infections (80, 81). This is more pronounced in fish without a choice to move to warmer water to fight off infection (108). The fish growth and survival could potentially be improved by providing equal access to resources, and thermal gradient, and enhancing feed with tryptophan and prebiotics/probiotics. Although the relationship between stress coping styles and immune functions in fish is not yet fully understood, research in this area is crucial for the aquaculture industry. Similarly, the interactions between the gut, brain, immune system, and gut microbiota are significant areas of study, particularly given the growing interest in alternative sources for fish feed production.

Social stress and the impact of different stress coping styles are significant factors to consider when using zebrafish and other teleosts as model organisms in biomedical research. The establishment of strong dominance hierarchies, particularly in low stocking densities, can lead to high levels of social stress and compromise the welfare of subordinate fish (28). This not only raises welfare concerns but also has the potential to alter the behaviour and physiology of the fish, ultimately affecting the results of the research. Therefore, maintaining optimal conditions for the fish is crucial in obtaining consistent and reliable results. Interestingly, social stress can also serve as a valuable model for studying the effects on behaviour and physiology, as demonstrated in the studies reviewed. In fact, social subordination in zebrafish could offer a valuable model for investigating depression and other affective disorders in humans.
